# Chaihuang-Yishen Granule Inhibits Diabetic Kidney Disease in Rats through Blocking TGF-β/Smad3 Signaling

**DOI:** 10.1371/journal.pone.0090807

**Published:** 2014-03-19

**Authors:** Ting Ting Zhao, Hao Jun Zhang, Xiao Guang Lu, Xiao Ru Huang, Wei Ku Zhang, Hua Wang, Hui Yao Lan, Ping Li

**Affiliations:** 1 Department of Pharmacology, Institute of Clinical Medical Sciences, China-Japan Friendship Hospital, Beijing, China; 2 Department of Medicine and Therapeutics, and Li Ka Shing Institute of Health Sciences, The Chinese University of Hong Kong, Hong Kong SAR; and CUHK Shenzhen Research Institute, Shenzhen, China; University of Tokushima, Japan

## Abstract

**Objective:**

Increasing evidence shows that TGF-β1 is a key mediator in diabetic nephropathy (DN) and induces renal fibrosis positively by Smad3 but negatively by Smad7. However, treatment of DN by blocking the TGF-β/Smad pathway remains limited. The present study investigated the anti-fibrotic effect of a traditional Chinese medicine, Chaihuang-Yishen granule (CHYS), on DN.

**Research Design and Methods:**

Protective role of CHYS in DN was examined in an accelerated type 1 DN induced by streptozotocin in uninephrectomized Wistar rats. CHYS, at a dose of 0.56 g/kg body weight, was administered by a daily gastric gavage for 20 weeks and the therapeutic effect and potential mechanisms of CHYS on diabetic kidney injury were examined.

**Results:**

Treatment with CHYS attenuated diabetic kidney injury by significantly inhibiting 24-h proteinuria and progressive renal fibrosis including glomerulosclerotic index, tubulointerstitial fibrosis index, and upregulation of extracellular matrix (collagen I, IV, and fibronectin), despite the same levels of blood glucose. Further studies revealed that inhibition of renal fibrosis in CHYS-treated diabetic rats was associated with inhibition of TGF-β1/Smad3 signaling as demonstrated by upregulation of Smad7 but downregulation of TGF-β1, TGF-β receptors, activation of Smad3, and expression of miRNA-21.

**Conclusions:**

CHYS may be a therapeutic agent for DN. CHYS attenuates DN by blocking TGF-β/Smad3-mediated renal fibrosis.

## Introduction

Diabetic nephropathy (DN) is one of the major microvascular complications of diabetes, and is the single most common cause of end-stage renal disease worldwide [1]. DN is characterized by excessive deposition of extracellular matrix (ECM) with thickening of glomerular and tubular basement membranes and increased amount of mesangial matrix, which ultimately leads to glomerulosclerosis and tubulointerstitial fibrosis accompanied by the development of albuminuria and a decline in renal function [2]–[4].

Transforming growth factor-β (TGF-β) signaling is a well-recognized pathway leading the development of DN [5]–[8]. It is well-established that after binding to its receptor, TGF-β1 signals through two critical downstream mediators, Smad2 and Smad3 (receptor-regulated Smads, R-Smads), to exert its biological activities such as ECM production, which is negatively regulated by Smad 7, an inhibitor Smad, by binding and degrading the phosphorylated type I receptor [9]–[11]. It is now clear that TGF-β/Smad3 mediates fibrosis by directly binding to many ECM promoters including collagen I, II, and III [12]–[15]. Moreover, recent studies also demonstrated TGF-β/Smad3-mediates renal fibrosis by upregulating microRNA-21 in both diabetic and non-diabetic kidney diseases [16]–[18].

Although a significant progress has been made in a better understanding of the pathogenesis of DN, treatment for diabetic kidney disease remains ineffective. In China, traditional Chinese medicine (TCM) is widely used for the treatment of diabetes and its complications, and has become a promising source of new therapeutic agents for DN [19]–[21]. Chaihuang-Yishen granule (CHYS, also called Qilong-Lishui granule) is formulated based on TCM theory for the treatment of chronic kidney disease. We reported previously that treatment with CHYS significantly reduces urinary protein excretion and serum creatinine in puromycin aminonucleoside-induced nephrotic syndrome in rats [22]. In the present study, we sought to determine if CHYS has therapeutic potential for DN and investigated underlying mechanisms of its action in rats with accelerated diabetic kidney.

## Materials and Methods

### Herbal Formulation and Components

CHYS was extracted from herbs, including root of *Astragalus membranaceus* (Fisch.) Bge. var. mongholicus (Bge) Hsiao., root of *Dioscorea nipponica* Mak., root of *Bupleurum chinense* DC., root of *Angelica sinensis* (Oliv.) Diels, leaf of *Pyrrosia petiolosa* (H. Christ) Ching, *Polyporus umbellatus* (Pers.) Fries, *Hirudo nipponica* Whitman in the ratio of 7∶5∶3∶3∶4∶4∶1 (W/W) on a dry-weight basis, respectively. The herbs were purchased from Beijing Tong Ren Tang Group Co. Ltd (Beijing, China). Preparation method of CHYS was described previously [22]. Dosage used in the experiment was based on our pilot study that a dose of 0.56 g/kg body weight produced the best result in inhibition of 24-h proteinuria in diabetic rats (Figure S1).

### Chromatographic Analysis of CHYS

**Figure 1:**
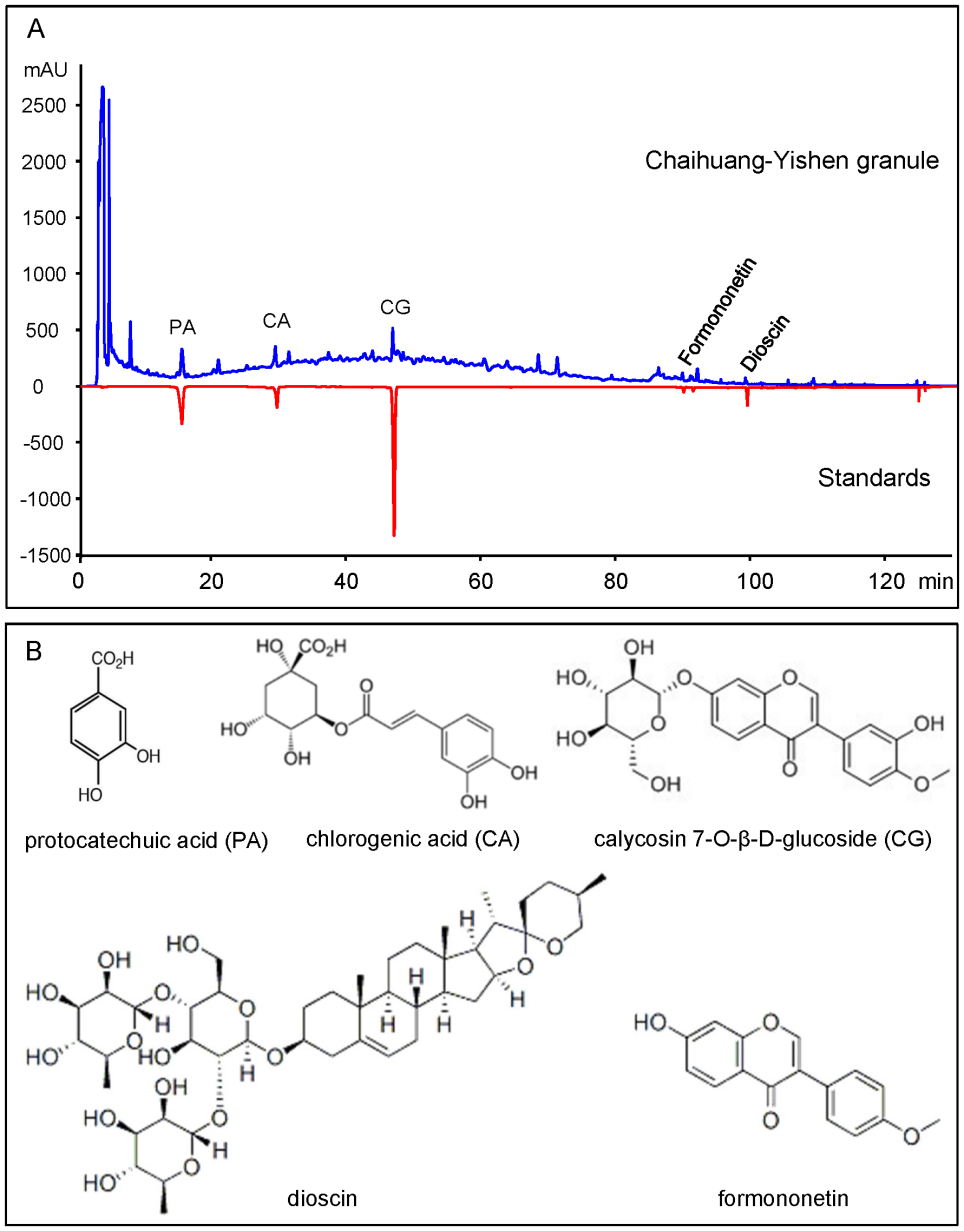
HPLC chemical fingerprint chromatogram of CHYS and chemical stuctures. HPLC chemical fingerprint chromatogram of CHYS including protocatechuic acid (PA), chlorogenic acid (CA), calycosin 7-O-β-D-glucoside (CG), formononetin and dioscin (below) in 210 nm (A). Chemical structures (B). Results show five major bioactive compounds in three batches of CHYS, including PA(0.424–0.434 μg/mg), CA (0.158–0.162 μg/mg), CG (1.702–1.738 μg/mg), formononetin (0.004–0.005 μg/mg), and dioscin (2.070–2.114 μg/mg).

CHYS was dissolved in methanol and filtered through a 0.45 μm filter (Microgen, Laguna Hills, CA, USA) before high performance liquid chromatography (HPLC) analyses. The HPLC system consisted of Agilent G1311A QuatPump, G1313A Auto-Sampler, and Agilent G1315B diode array detector. HPLC analysis was performed using a Phenomenex Luna C18 column (4.6×250 mm, particle size 5 μm) with acetonitrile (as Solvent A): 0.5% phosphoric acid (as Solvent B) as mobile phase at a flow rate of 1.0 mL/min at the column temperature of 30°C. A linear gradient elution was applied from 5% of Solvent A starting from 0 to 10 min, 5–30% of Solvent A starting from 10 to 80 min, 30–100% of Solvent A starting from 80 to 120 min. Pure standards including protocatechuic acid (PA), chlorogenic acid (CA), calycosin 7-O-β-D-glucoside (CG), formononetin and dioscin were purchased from the National Institutes for Food and Drug Control (Beijing, China) and were used as external standards in the HPLC analysis. Identification of HPLC peak fractions was carried out by comparing retention times and UV spectra with the standards. Five major bioactive compounds in the three batches of CHYS included PA (0.424–0.434 μg/mg), CA (0.158–0.162 μg/mg), CG (1.702–1.738 μg/mg), formononetin (0.004–0.005 μg/mg), and dioscin (2.070–2.114 μg/mg) (Figure 1).

### Experimental Model of Diabetes in Rats

Male Wistar rats weighing 200±20 g were purchased from Beijing HFK Bio-Technology Co. Ltd. (Beijing, China, Certificate No. SCXK 2002-0010) and randomly divided into sham group and diabetic group. To accelerate the development of diabetic kidney disease, rats in the diabetic group underwent right uninephrectomy. Sham-control rats received sham-operation consisting of laparotomy and manipulation of the renal pedicles but without damage to the kidney as previously described [23]. One week after uninephrectomy, diabetes was induced by a single intraperitoneal injection of streptozocin (STZ, Sigma-Aldrich, St Louis, MO) at a dose of 40 mg/kg diluted in the citrate buffer (0.1 mol/L, pH 4.0). Seventy-two hours after STZ injection, 42 rats developed hyperglycemia with blood glucose levels over 16.7 mmol/L. All diabetic rats were randomly assigned to three groups and treated with CHYS or vehicle control, or fosinopril (an ACE inhibitor as known positive control). In CHYS-treated rats (*n* = 14, CHYS), a daily gavage at a dose of 0.56 g/kg body weight was given for 20 weeks, while diabetic animals that received vehicle control without CHYS were used as negative treatment control (*n* = 14, DN). In addition, diabetic rats (*n* = 14) treated with daily fosinopril at a dose of 1.60 mg/kg body weight were used as positive treatment controls. A group of 10 rats that received sham-operation without STZ was used as normal controls. Blood glucose was measured every four weeks by One Touch Ultra blood glucose monitoring system (LifeScan Inc., Milpitas, CA, USA) by tail-vein blood sampling. Rats were housed individually in metabolic cages (Fengshi Inc., Suzhou, China) for 24-h urinary collection at 4-week intervals. Urinary protein was assessed by the Bradford method. All animals were housed at a temperature of 20–25°C, humidity of 65–69%, and were subjected to a 12-h light/dark cycle with free access to food and tap water. After induction of diabetes, all rats were euthanized at week 20 after induction of diabetes. The study protocol was approved by the Ethics Committee of China-Japan Friendship Institute of Clinical Medical Sciences and performed in accordance with the NIH Guiding Principles for *the Care and Use of Laboratory Animals* (No. 2010-A10).

### Histology and Immunohistochemistry

Kidney tissues were fixed in 10% phosphate buffered formalin solution and embedded in paraffin. Paraffin sections (2–3 μm ) were stained with periodic acid silver methanamine (PASM), periodic acid-Schiff (PAS), and Masson’s trichrome. The degree of glomerulosclerosis, defined as ECM depositon and mesangial expansion, was evaluated at 40× power for 20 cortical fields using the scoring system (0–4 grades) as previously described [3]. Tubulointerstitial damage was assessed at 20× power-field using the scoring system (1–6 grades) [24]. Briefly, the percentage of affected areas in 10-cortical fields under 20× power was evaluated. The degree of tubulointerstitial damage including tubular dilation, tubular atrophy, cast formation, and interstitial mononuclear cell and extracellular matrix accumulation (interstitial volume) was scored (1 =  less than 10%; 2 = 10–25%; 3 = 26–50%, 4 = 51–75%, 5 = 76–95%, 6 =  more than 95%).

Immnunohistochemistry was performed on paraffin sections (3-μm) using a microwave-based antigen retrieval technique [25]. Antibodies used in this study included: primary antibodies against fibronectin and phosphorylated Smad2/3 (Santa Cruz Biotechnology Inc., Santa Cruz, CA, USA), collagen I and IV (SouthernBiotech, Birmingham, AL, USA). After being immunostained with the secondary antibodies, sections were developed with diaminobenzidine to produce a brown product and counterstained with hematoxylin. Quantitation of immunostaining was carried on coded slides as previously described [26], [27]. Accumulation of collagen I and fibronectin was determined using the quantitative Image Analysis System (Image-Pro Plus v 6.0, Media Cybernetics, Warrendale, PA, USA). Briefly, ten random fields in both glomeruli and tubulointerstitium under 20× power were outlined and positive staining patterns were identified. Then, the percentage of positive area in the examined field was measured. Accumulation of collagen IV in twenty random glomeruli under 40× power were analyzed as above. The arterial lumen space was excluded from the study. Data were expressed as percentage of positive area examined. In addition, the number of phospho-Smad2/3 was counted in twenty random glomeruli and expressed as cells/glomerular cross-section, whereas positive cells in the tubulointerstitium were counted in twenty random fields for each sample under high-power fields (40×) by means of a 0.0625-mm^2^ graticule fitted in the eyepiece of the microscope, and expressed as cells per mm^2^ [11]. Data were expressed as the mean ± SE. All counting was performed on blinded slides.

### RNA Extraction and Quantitative RT-PCR Analysis

Renal cortex was collected by carefully removing the renal pelvis and medullar tissues and was frozen at −80°C for analysis of the gene of interest. Trizol reagent (Invitrogen, Life Technologies, Carlsbad, CA, USA) was used to isolate total RNA from kidney tissues following the manufacturer’s instructions. Template cDNA was prepared using reverse transcriptase. MicroRNA-21 expression in individual samples was quantified by real-time PCR with the TaqMan MicroRNA Assay (Applied Biosystems, Foster City, CA, USA) as described previously [27]. U6 was used as an internal standard, and the ratio of microRNA-21 was normalized with U6. Each sample was run in duplicate and results from groups of 14 samples were statistically analyzed as described below.

Primers used for detection of rat mRNAs were: collagen IV: forward 5′-GGCGGTGCACAGTCAGACCAT-3′ and reverse 5′-GGAATAGCCAATCCA CAGTGA-3′; collagen I: forward 5′-CACAAGCGTGCTGTAGGTGA-3′ and reverse 5′-TGCCGTGACCTCAAGATGTG-3′; fibronectin: forward 5′-GAGGAGGTCCAAATCGGTCATGTT-3′ and reverse 5′-AACTGTAAGGGCTCTTCGTCAATG-3′; TGF-β1: forward 5′-ACGTCAGACATTCGGGAAGCAGTG-3′ and reverse 5′- GCAAGGACCTTGCTGTACTGTGTG-3′; Smad7: forward 5′-AGAAGATATCCAGGGAGGGCTCTT-3′ and reverse 5′-CGCTGTACCTTCCTCCGAT-3′; Smad3: forward 5′-CTCCTACTACGAGCTGAACCAG-3′ and reverse 5′-CTCATGCGGATGGTGCACATTC-3′; β-actin: forward 5′-GAGACCTTCAACACCCAGCC-3′ and reverse 5′-GCGGGGCATCGGAACCGCTCA-3′. Levels of mRNA expression were subjected to house keeping gene β-actin and expressed as the mean ± SE.

### Western Blot Analysis

Renal cortical tissues were lysed with radioimmunoprecipitation assay (RIPA) buffer, and proteins were extracted for Western blot analysis as described previously [26]. Antibodies used in this study included primary antibodies against fibronectin, TGF-β1, TGF-β receptor I (TβRI), phospho-TGF-β receptor I (p-TβRI), TGF-β receptor II (TβRII), and Smad7 (Santa Cruz Biotechnology, Santa Cruz, CA, USA), Smad3 (Zymed Laboratories, South San Francisco, CA, USA) and phospho-Smad3 (p-Smad3, Cell Signaling Technology Inc., Danvers, MA, USA), collagen I and IV (SouthernBiotech, Birmingham, AL, USA), and IRDyeTM800 conjugated secondary antibodies (Rockland Immunochemicals Inc., Gilbertsville, PA, USA). Signals were detected with Odyssey Infrared Imaging System (LI-COR Biosciences, Lincoln, NE, USA) and quantitated with Image J program (National Institutes of Health, Bethesad, MD, USA). Ratio for the protein examined was normalized against β-actin and expressed as mean ± SE.

### *In Situ* Hybridization

5′-digoxigenin (DIG) and 3′-DIG labeled antisense-locked nucleic acid oligonucleotides for hsa-microRNA-21 5′-3′(/5DigN/−TCAACATCAGTCTGATAAGCTA/3 Dig_N/) and U6, hsa/mmu/rno, 5′-3′(GTGTAACACGTCTATACGCCCA) as a negative control were purchased from Exiqon (Vedbaek, Denmark). Procedure for *in situ* hybridization was performed as previously described [16]. In brief, 6-μm slides were prepared from formalin-fixed, paraffin-embedded kidney tissues. After deparaffinization and deproteinization (15 μg/mL) for 10 minutes at 37°C, slides were dehydrated in gradient ethanol and hybridized with DIG-antisense microRNA-21 probe (30 nM) at 53°C in a 1× hybridization buffer for 1 h. After being washed, slides were blocked and incubated with alkaline phosphatase-conjugated anti-DIG Fab fragments (1∶800; Roche Applied Science, Indianapolis, IN, USA) and visualized for color detection.

### Statistical Analysis

Data were expressed as the mean ± SE and analyzed using one-way ANOVA followed by post comparision with the Newman-Keuls program from GraphPad Prism 5.0 (GraphPad Software, San Diego, CA, USA).

## Results

### CHYS Treatment Attenuates Proteinuria and Histological Damage in Diabetic Rats

**Figure 2:**
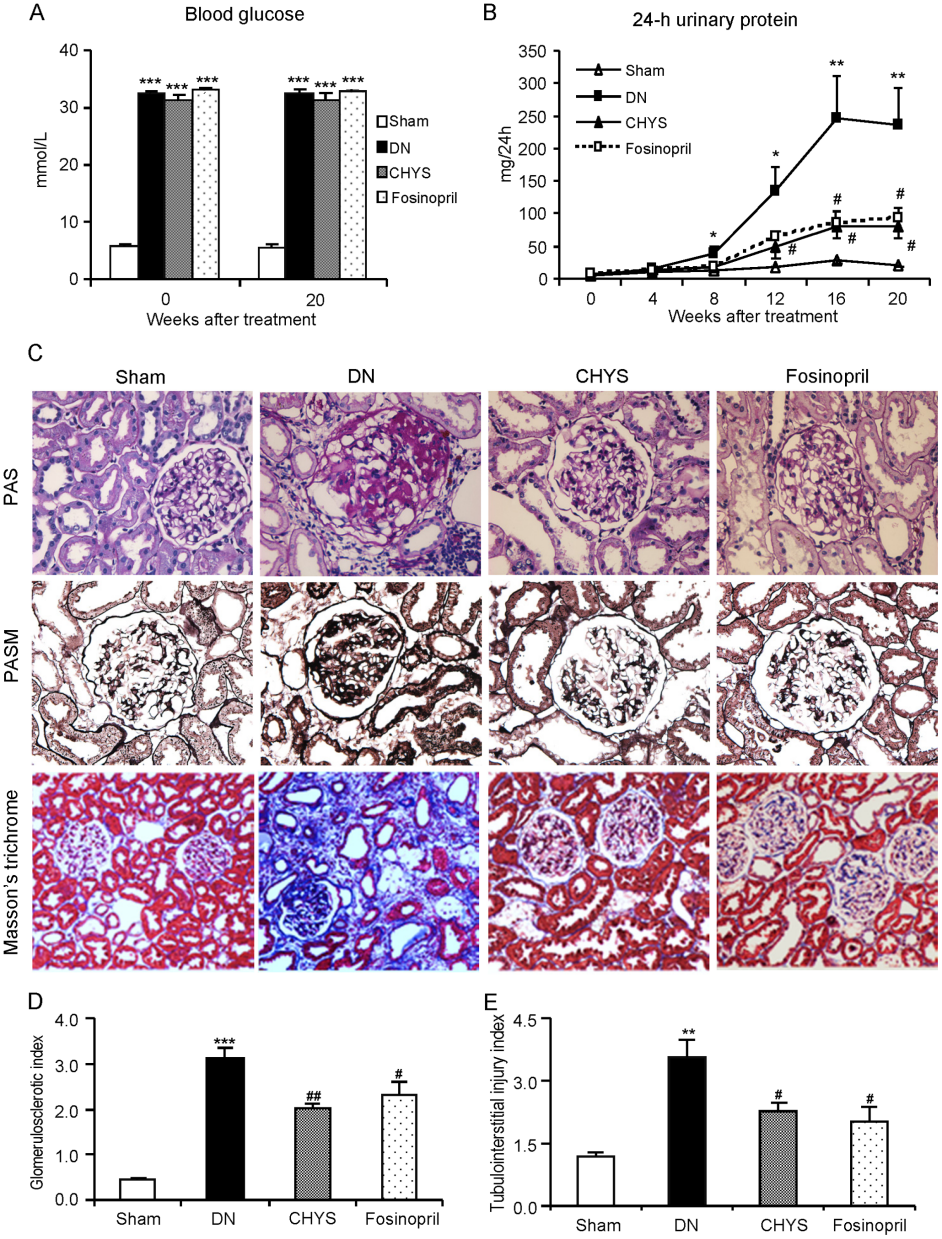
CHYS attenuates renal injury in diabetic rats. 24-h urinary protein (A). Blood glucose (B). Periodic acid schiff (PAS, magnification×400), periodic acid silver methanamine (PASM, magnification×400), and Masson’s trichrome-stained (Magnification×200) sections (C). Glomerulosclerotic index (D). Tubluointerstitial injury index (E). Data represent groups of 14 rats and are expressed as mean ± SE. *P<0.05, **P<0.01, ***P<0.001 *vs*. sham group; ^#^ *P*<0.05, ^##^ *P*<0.01 *vs*. DN group.

All rats under experiment developed hyperglycemia with high levels of blood glucose over the 20-week study period (Figure 2A). A significant increase in 24-h proteinuria was evidenced in DN rats at week 8 after STZ injection and peaked over 16–20 weeks (Figure 2B). Treatment with either CHYS or fosinopril had no effect on blood glucose levels (Figure 2A), but largely reduced 24-h proteinuria over 12–20 weeks equally when compared to the DN rats (Figure 2B).

Morphologically, staining with PAS, PASM and Masson-trichrome revealed that diabetic rats without treatment developed a moderate expansion in mesangial matrix, thickening of glomerular basement membrane, glomerulosclerosis, tubular atrophy, and extracellular matrix deposition in tubulointerstitium at week 20 (Figure 2C). In contrast, treatment with either CHYS or fosinopril for 20 weeks significantly inhibited these histological damages equally (Figure 2D–E).

### CHYS Treatment Inhibits Renal Fibrosis in Diabetic Rats

**Figure 3:**
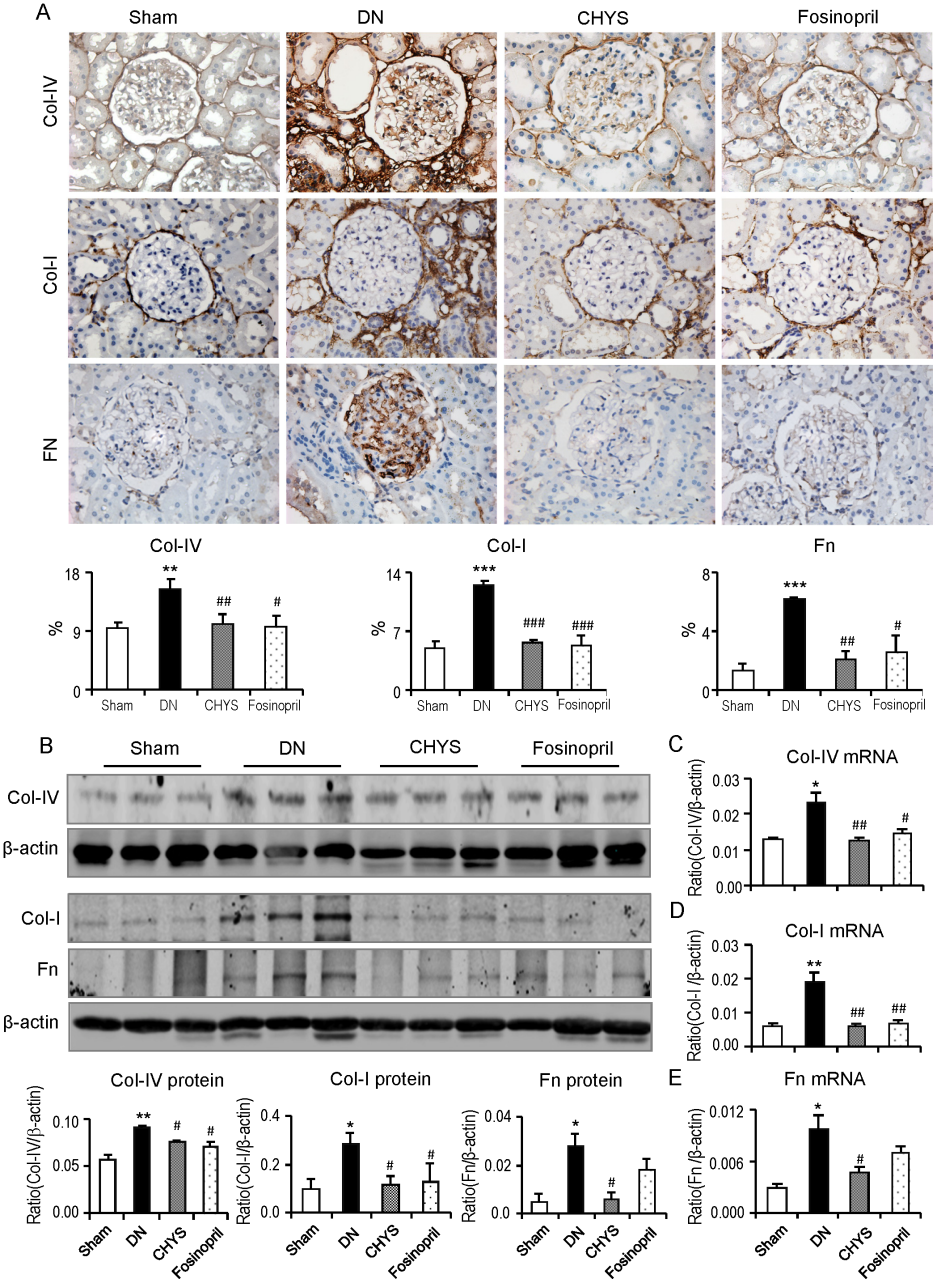
CHYS inhibits renal fibrosis in diabetic rats. Immunohitochemistry and quantitative analysis (A), Western blots and quantitative analysis (B), Real-time PCR for Collegen IV (C), collagen I (D) and fibronectin (E) expression, respectively. Data represent mean ± SE for groups of 14 rats. *P<0.05, **P<0.01, ***P<0.001 *vs*. sham rats; ^#^ *P*<0.05, ^##^ *P*<0.01, ^###^ *P*<0.001 *vs*. DN group. Collegen I: Col-I. Collegen IV: Col-IV. Fibronectin: Fn.

Immunohistochemistry, Western blot and real-time PCR analysis showed that compared with sham-control rats, moderate renal fibrosis developed in the diabetic kidney as demonstrated by a significant accumulation of collagen I, collagen IV, and fibronectin in the renal cortex (Figure 3). All of these fibrotic changes in the diabetic kidney were largely attenuated by treatment with either CHYS or fosinopril (Figure 3).

### Rebalancing the TGF-β/Smad3 Signaling Is a Key Mechanism by Which CHYS Inhibits Diabetic Renal Fibrosis

**Figure 4:**
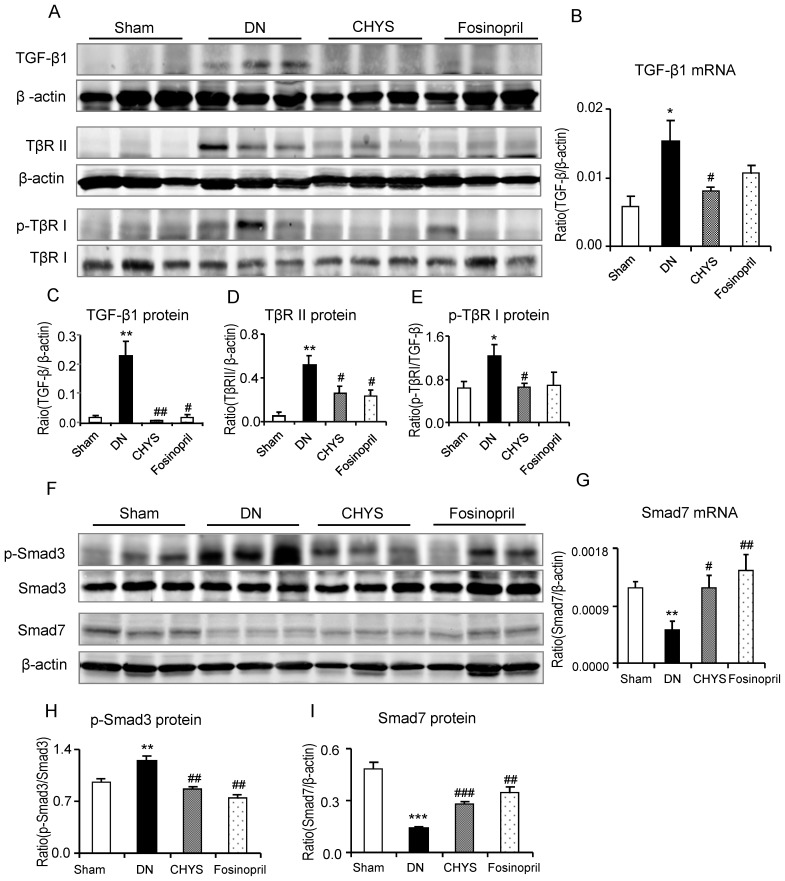
CHYS blocks activation of the TGF-β/Smad signaling pathway in the diabetic rats. Western blot (A) and quantitative analysis of renal TGF-β (C), TβR II (D), p-TβR I and TβR I (E) expression, respectively. Real-time PCR for renal TGF-β1 mRNA expression (B). Western blot (F) and quantitative analysis of phospho-Smad3, Smad3 (H) and Smad7 (I) expression, respectively. Real-time PCR for renal Smad7 mRNA expression (G). Data represent mean ± SE for groups of 14 rats. *P<0.05, **P<0.01, ***P<0.001 *vs*. sham group; ^#^ *P*<0.05, ^##^ *P*<0.01, ^###^ *P*<0.001 *vs*. DN group.

**Figure 5:**
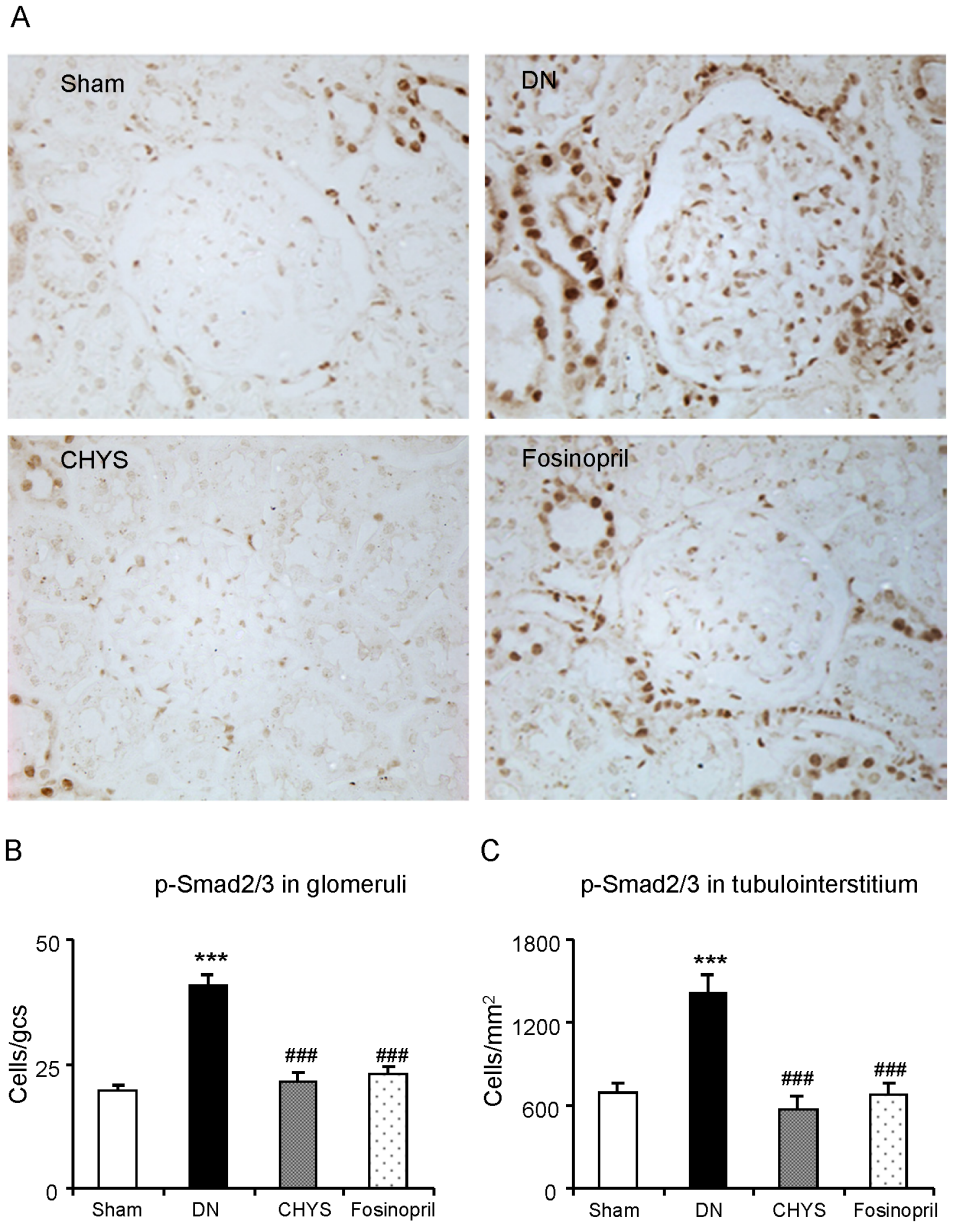
CHYS inhibits activation of Smad signaling in the diabetic kidney. Phosphorylated Smad2/3 (p-Smad2/3) nuclear location (A. Magnification×400). Quantitative analysis of nucleated p-Smad2/3 in glomeruli (B) and tubulointerstitium (C) respectively. Data represent mean ± SE for groups of 14 rats. *P<0.05, **P<0.01, ***P<0.001 *vs*. sham group; ^#^ *P*<0.05, ^##^ *P*<0.01, ^###^ *P*<0.001 *vs*. DN group.

We next investigated the mechanisms by which CHYS treatment attenuated diabetic renal fibrosis by studying the TGF-β/Smad signaling pathway. TGF-β1/Smad3 signaling was highly activated in the diabetic kidney as revealed by a marked upregulation of TGF-β1 and TβRII and higher levels of p-TβRI and p-Smad3, but lower levels of an inhibitory Smad7 in both mRNA and protein expression levels (Figures 4 and 5). Treatment with CHYS significantly blocked a marked upregulation of TGF-β1, TβRII, and phosphorylation of TβRI and Smad3, which was associated with a significant upregulation of renal Smad7 (Figures 4 and 5). Similar results were observed in diabetic rats treated with fosinopri, although expression of TGF-β1 mRNA and phosphorylation levels of TβRI remained high (Figures 4 and 5).

**Figure 6:**
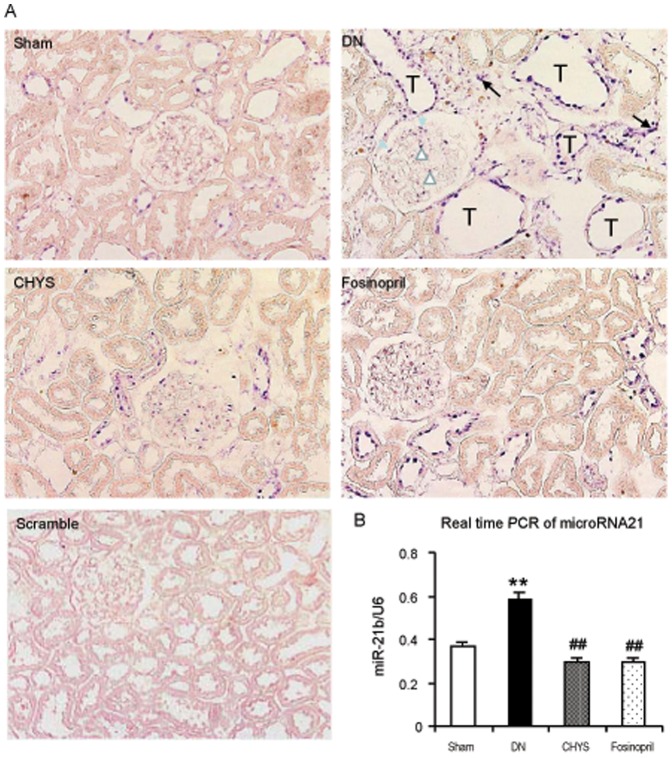
CHYS downregulates microRNA-21 expression in the kidney of diabetic rats. *In situ* hybridization (A. Magnification×200) and Real-time PCR (B) for microRNA-21 expression. Note that examples of microRNA-21 expression by mesangial cells (opened arrowheads), podocytes (closed arrowheads), tubular epithelail cells (T), and interstitial fibroblasts (arrows) are illustrated in DN panel. Data represent mean ± SE for groups of 14 rats. *P<0.05, **P<0.01 *vs*. sham rats; ^#^ *P*<0.05, ^##^ *P*<0.01 *vs*. DN rats.

Because it has been reported that microRNA-21 is a downstream of TGF-β/Smad3 and plays a pathogenic role in renal fibrosis in both diabetic and non-diabetic kidney diseases [16], [18]. We then examined the expression of microRNA-21 in the diabetic kidney treated with or without CHYS or fosinopril. *In situ* hybridization revealed that expression of microRNA-21 was significantly upregulated in the diabetic kidney in both glomeruli and tubulointerstitium, presumably by mesangial cells, podocytes, tubular epithelial cells, and interstitial fibroblasts, which were largely inhibited by treatment with CHYS or fosinopril (Figure 6A). Quantitative real-time PCR confirmed this finding (Figure 6B).

## Discussion

Traditional Chinese medicine has long been used to treat chronic kidney diseases, including DN. In this study, we examined the therapeutic effect and the underlying mechanisms of a TCM remedy, CHYS, in a rat model of DN. We found that administration of CHYS significantly inhibited STZ-induced kidney fibrosis and markedly decreased proteinuria. The inhibitory effect of CHYS on diabetic kidney disease was associated with inactivation of TGF-β/Smad3 signaling.

The most significant finding of this study was that CHYS inhibited renal fibrosis in the diabetic kidney by rebalancing TGF-β/Smad signaling. It is well established that the binding of TGF-β1 to TβRII can activate TβRI-kinase, resulting in phosphorylation of Smad2 and Smad3. Subsequently, phosphorylated Smad2 and Smad3 bind to the common Smad4 and form the Smad complex to translocate into the nucleus to regulate the target gene transcription [28]. Smad7, an inhibitory Smad, can block TGF-β/Smad signaling by binding to Smurf2 to form an E3 ubiquitin ligase that targets the TGF-β receptor as well as Smads including Smad7 for degradation [29]. Once Smad7 is degraded, activation of Smad3 and renal fibrosis is enhanced. In the context of DN, TGF-β/Smad3 signaling is highly activated, which is associated with downregulation of renal Smad7, resulting in renal fibrosis as seen in both experimental and human diabetic kidneys [11], [30] and *in vitro* under high glucose and advanced glycation end products conditions [30], [31]. The functional importance of TGF-β/Smad signaling in DN is demonstrated in a number of animal models in which deletion of Smad3 or overexpression of Smad7 inhibits diabetic renal injury [11], [32], [33]. In the present study, STZ-induced DN was associated with a marked activation of Smad3 but a loss of Smad7, suggesting the imbalance between Smad3 and Smad7 signaling in the pathogenesis of DN. In contrast, treatment with CHYS attenuated diabetic renal injury by rebalancing the TGF-β/Smad signaling pathway. A similar mechanism was also evident by a positive treatment control with fosinopril. Consistent with a critical role for angiotens in II in DN [34], [35], blockade of angiotensin inhibited activation of TGF-β/Smads signaling. Thus, blockade of TGF-β/Smad3-mediated renal fibrosis could be an important mechanism by which CHYS attenuated diabetic kidney disease.

Increasing evidence show that upregulation of microRNA-21 plays a critical role in renal fibrosis including DN [36], [37]. It is now clear that microRNA-21 is regulated by TGF-β/Smad3 and acts as a downstream mediator of TGF-β/Smad3-driven renal fibrosis [16]. During renal fibrosis, microRNA-21 is upregulated in both diabetic and non-diabetic kidney disease [16], [18]. We have previously shown that Smad3 binds the Smad binding site located in the microRNA-21 promoter and induces pri-microRNA-21 transcription [16]. MicroRNA-21 in turn promotes TGF-β/Smad3 signaling by repressing Smad7 [18], [38]. Thus, microRNA-21 functions in a feed-forward loop that leads to enhanced TGF-β/Smad3 signaling [18], [38]. In the present study, treatment with CHYS was able to downregulate renal microRNA-21, which may result in upregulation of renal Smad7 through which activation of TGF-β/Smad3 and Smad3-dependent microRNA-21 expression were inhibited.

## Conclusion

In conclusion, the present study demonstrates that CHYS may be a novel therapeutic agent for DN. Blockade of TGF-β/Smad3-mediated renal fibrosis by downregulating TGF-β1 and microRNA-21 expression, thereby restoring the balance of Smad signaling by upregulating an inhibitory Smad7, resulting in a possible underlying mechanism by which CHYS inhibits diabetic nephropathy associated with fibrosis.

## Supporting Information

Figure S1 **CHYS attenuates 24-hour urine protein in a dosage-dependent manner in diabetic rats.** CHYS-L: 0.56 g/Kg body weight, CHYS-M: 1.12 g/Kg body weight, CHYS-H: 2.24 g/Kg body weight. Data represent mean ± SE for groups of 14 rats. *P<0.05, **P<0.01 *vs*. sham rats; ^#^ *P*<0.05 *vs*. DN rats; ^Δ^ *P*<0.05 *vs*. CHYS-H; ^@^ *P*<0.05 *vs*. CHYS-M.
(TIF)
